# “Caregiving is like on the job training but nobody has the manual”: Canadian caregivers’ perceptions of their roles within the healthcare system

**DOI:** 10.1186/s12877-021-02354-z

**Published:** 2021-06-30

**Authors:** Susan Law, Ilja Ormel, Stephanie Babinski, Kerry Kuluski, Amélie Quesnel-Vallée

**Affiliations:** 1 Trillium Health Partners – Institute for Better Health, 100 Queensway West, 6th Floor CA Building, Mississauga, ON L5B 1B8 Canada; 2 University of Toronto – Institute for Health Policy, Management and Evaluation, 155 College Street, 4th Floor, Toronto, ON M5T 3M6 Canada; 3 St. Mary’s Research Centre, 3830 avenue Lacombe, Montreal, QC H3T 1M5 Canada; 4 Department of Family Medicine, McGill University, 5858 Cote-des-Neiges Road, Montreal, QC H3S 1Z1 Canada; 5 Ryerson University, Faculty of Community Services, 350 Victoria Street, Toronto, ON M5B 2K3 Canada; 6 ELLICSR Health, Wellness & Cancer Survivorship Centre, Department of Supportive Care, Princess Margaret Cancer Centre, University Health Network, 585 University Ave, Toronto, ON M5G 2N2 Canada; 7 Department of Epidemiology, Biostatistics and Occupational Health, McGill University, 1020 Pine Avenue West, Montreal, QC H3A 1A2 Canada

**Keywords:** Caregiving, Carers, Canada, Healthcare system, Qualitative research, Chronic illness

## Abstract

**Background:**

Stepping into the role of an unpaid caregiver to offer help is often considered a natural expectation of family members or friends. In Canada, such contributions are substantial in terms of healthcare provision but this comes at a considerable cost to the caregivers in both health and economic terms.

**Methods:**

In this study, we conducted a secondary analysis of a collection of qualitative interviews with 39 caregivers of people with chronic physical illness to assess how they described their particular roles in caring for a loved one. We used a model of caregiving roles, originally proposed by Twigg in 1989, as a guide for our analysis, which specified three predominant roles for caregivers – as a resource, as a co-worker, and as a co-client.

**Results:**

The caregivers in this collection spoke about their roles in ways that aligned well with these roles, but they also described tasks and activities that fit best with a fourth role of ‘care-coordinator’, which required that they assume an oversight role in coordinating care across institutions, care providers and often advocate for care in line with their expectations. For each of these types of roles, we have highlighted the limitations and challenges they described in their interviews.

**Conclusions:**

We argue that a deeper understanding of the different roles that caregivers assume, as well as their challenges, can contribute to the design and implementation of policies and services that would support their contributions and choices as integral members of the care team. We provide some examples of system-level policies and programs from different jurisdictions developed in recognition of the need to sustain caregivers in their role and respond to such limitations.

## Background

Caregivers who provide unpaid care for family or friends with an illness or disability, occupy an uneasy position within the healthcare system. The caregiver contribution may be formally acknowledged if taken into account as part of the care recipient’s needs assessments and service allocation. It is, however, often taken for granted by societies and individuals as a ‘natural’ expectation for what families do and with caregivers often relegated to the margins of care teams, without training or support [1]. The ‘gift’ of caregivers’ contributions in terms of the care they provide for loved ones and the economic savings for the healthcare system [2] often comes with personal consequences that include poor physical and mental health, social isolation, professional sacrifices and economic loss [3–7]. Studies of caregivers’ roles in healthcare systems refer to ‘hidden costs’ and ‘invisible contributions’ [8], and the need for attention to caregivers’ wellbeing and support [9]. Caregivers themselves have highlighted the personal value and benefits they realize in caring for another person, yet given that caregivers’ contributions will only grow in importance, it is imperative to develop a more balanced equation through policies and programs that aim to support them in their role [10, 11].

The prevailing conceptual model of the role of caregivers was developed more than a quarter century ago [12], based on a system’s perspective, with only minor adjustments over time [13]. Since then, there have been significant changes in health, healthcare, and social structures in most developed systems, with implications for caregivers’ roles. The epidemiological profile of the population in need of care has changed substantially due to technical advances, such as the introduction of new medical and surgical interventions, enhancing survival rates among patients with chronic conditions, but with increasing prevalence of frailty and multi-morbidity. One study reported that, the prevalence of two or more chronic conditions rose by 29% in Canada, among adults aged 40 years and older, between 2001 and 2012 [14]. Yet current clinical systems that evolved from specialist care and single, disease-oriented programs are poorly equipped to deal with such multi-morbidity [15]. It has been argued that person-centered or relationship-centred approaches may be more effective in this evolving scenario [16–18], but also that informal caregivers should feature more centrally in the provision of individual care and in decision-making [19, 20]. Societal changes have also had an impact on the nature of caregiving, given, for example, smaller families, more women in the workforce, and higher divorce rates, which all reduce the number and proximity of family members available to care [21]. Collectively, these changes have contributed to the increasing care demands of persons cared for by informal caregivers in the current medical and social context.

Our objectives were to explore how caregivers espoused ideas about their roles in consideration of Twigg’s typology, and to examine their perceptions of barriers in performing these roles. We have drawn from qualitative interviews conducted previously by our team involving a diverse sample of caregivers caring for people with chronic physical illness in Canada. We revisit this uneasy balance between assumptions of caregiving as a ‘natural’ expectation and the increasingly complex context of caring for someone at home or from afar.

### Caregivers’ roles from the system’s perspective: revisiting the typology

In 1989, Twigg proposed a seminal model that articulated three roles for caregivers, as viewed from the perspective of social care agencies: caregiver as a resource, co-client, and co-worker [12]. This typology has been subject to subsequent iterations and alternate frameworks have emerged, for instance, in consideration of a ‘superseded carer’ where agencies assume responsibility to liberate the care recipient from a caregiving relationship [13], or to consider different professional perspectives [22], illnesses [23], patient populations [24], and health system contexts [25, 26]. These efforts have, however, left the original typology fundamentally unchallenged and substantially unchanged. In this paper, we consider Twigg’s original model, describing three roles for caregivers (described briefly below), as a framework to help guide our analysis of qualitative data in this study, to assess fit and explore any gaps in its application to the caregiving narratives about roles as shared in our original study.

#### Caregiver as a resource

This is the most common view of caregivers as portrayed through different studies. The role as ‘resource’ is presented as “being spontaneous and improvised”, “motivated by love and obligation”, where caregivers possess the “necessary skills, knowledge and competence” ([25], p.31]. These quotes reflect the prevailing view (in North America at least) that informal care is not a substitute for formal care, but merely fills in the gaps [25]. As a ‘given’ resource [12], this notion reinforces an implicit social norm of caregiving as a ‘natural’ adoption of tasks related to social responsibilities assumed as parents, children or partners.

#### Caregiver as a co-worker

In describing the caregiver as a co-worker, authors evoke “a co-operative and enabling role”, where the “caregiver and healthcare professionals work in parallel with each other” ([12], p.58), as “equal players in the caregiving process” ([25], p.33). Somewhat tempering this ideal, however, Ward-Griffin and McKeever notice that nurses recognized caregivers’ expertise but, under the notion of ‘teamwork’, collaborated “in an essentially co-opting and controlling way” ([22], p.96]. Viewed from the caregiver’s perspective, Stajduhar et al. describe caregivers as having “established and interpreted themselves as integral members of the end-of-life caregiving team”, thus validating this role as being salient to their experience ([23], p.1794].

#### Caregiver as a co-client

As co-clients, the system acknowledged that caregivers themselves are in need of care, requiring professional support to cope with the situation and function optimally in their role [12, 22, 25]. This view is frequently espoused by caregivers themselves, for instance, in a study [23] where caregivers of palliative care patients saw themselves as being in need of care together with the care recipient and were actively seeking support.

## Methods

In the original qualitative descriptive study [27], we conducted in-depth individual interviews with a diverse sample of adult caregivers from across Canada caring for someone with a chronic physical illness. Interviews were audio and/or video recorded. Diversity was considered on two dimensions: caregivers’ individual characteristics (e.g. age, sex, ethnicity, socioeconomic status) and attributes of the caregiving experience (e.g. chronic condition of the care recipient, years of caregiving). The original study including the methods have been published [6].

Ethics approval for the original study and secondary analysis was obtained from St. Mary’s Hospital Research Ethics Committee. Patient participants provided informed consent for the original interview and, following review of their transcript, provided a second consent for use of their interview material for the publication of results on our web site (www.healthexperiences.ca), secondary analysis, teaching, and academic publication. Participants could choose to use their own name or an alias as part of their consent for use of their data in publications or secondary research; these are the names that appear with the selected quotes in the results section.

We interviewed 41 participants between September 2011 and October 2012 across seven Canadian provinces; two interviews were with couples – one couple caring for their adult child and another caring for an aging parent – and one participant was interviewed twice at her request. Three participants withdrew for unknown reasons, leaving 39 participants, and 40 transcripts for analysis. The characteristics of participants are presented in Table 1. The overall approach for the analysis and key themes were reviewed together with an expert panel including caregivers and community-based organizations.

**Table 1:** Characteristics of 39 participants

Age at Start of Caregiving Duties (years), ***n*** = 39	N	%	Current Age (years), ***n*** = 39	N	%
< 20	3	7.7	20–39	5	12.8
20–39	13	33.3	40–59	14	35.6
40–59	22	56.4	60–79	18	46.2
60+	1	2.6	80–89	2	5.1
**Caregiver Status,** ***n*** = 43^*^			**Caregiver Sex,** ***n*** **= 39**		
Current Caregiver	32	74.4	Female	28	71.8
Post-caregiver	11	25.6	Male	11	28.2
**Time Spent Caregiving (Years),** ***n*** **= 39**			**Age of Care Recipient (years),** ***n*** **= 43**^**1**^		
0–9	14	35.0	< 20	1	2.3
10–19	15	38.5	20–39	2	4.7
20–29	6	15.4	40–59	8	18.6
30–39	4	10.3	60–69	12	27.9
40–49	0	0	70+	11	25.6
50–59	1	2.6	Deceased	9	23.1
**Care Recipient Designation,** ***n*** = 43^*^			**Caregiver Employment,** ***n*** **= 39**		
Mother	12	27.9	Full-time	12	30.8
Father	2	4.7	Part-time	8	20.5
Wife	5	11.6	Retired	13	33.3
Husband	17	39.5	Lost job due to caregiving duties	1	2.6
Friend	3	7.0	Sick leave	1	2.6
Child	4	9.3	Not working	4	10.3
**Care Recipient Residence,** ***n*** **= 39**			**Marital Status,** ***n*** **= 39**		
Another household	1	2.6	Married	27	69.2
Same household	23	59.0	Widowed	5	12.8
Health care institution	6	15.4	Separated	1	2.6
Deceased	9	23.1	Single	6	15.4
**Mother Language, n = 39**			**Ethnic Background, n = 39**		
English	26	66.7	Aboriginal	2	5.1
French	4	10.3	Asian	2	5.1
Bilingual	4	10.3	Black	1	2.6
Other	5	12.8	Caucasian	34	87.2

* *N* = 43 as some caregivers took care of, or had cared for, more than one person

For this study, we conducted a secondary qualitative analysis of the original transcripts to explore caregivers’ perspectives on their roles more specifically. We used an iterative inductive-deductive approach. First, we undertook thematic analysis to identify emergent themes related to how people spoke about their roles [27], using the method of constant comparison involving ongoing review of emergent and existing findings in the analysis [28, 29]. Second, we organized role descriptions within the text as per the Twigg framework. The transcripts were reviewed by one researcher to identify initial codes from the data (e.g. activities related to proving care) and general categories (e.g. grey areas of responsiblities between healthcare professionals and caregivers); a second researcher worked together with the first to refine the categories, and select data and quotes pertinent to the emergent themes. The Twigg framework was applied in a deductive approach to consider the findings in terms of fit with the three different roles (e.g. challenges for caregivers as co-worker), and the researchers identified and discussed any outlying descriptions of roles for further analysis and interpretation. Differences in coding and interpretation were discussed and resolved between the two researchers. Analysis was conducted by IO and SL and discussed with AQ. The researchers adopted an open, reflective approach to the analysis, with consideration of their own personal experiences of caregiving and as women in discussions of the interpretations of the data.

## Results

The results of our analysis are presented below in terms of the extent to which caregivers’ experiences and perceptions of their tasks and activities align with the three roles identified in Twigg’s model. We also present the articulation of a fourth role for further consideration. Although the findings related to each role are described separately below, caregivers commonly described the fulfillment of multiple roles throughout their day-to-day activities. We also present the limitations or challenges they described in fulfilling these particular roles.

### Caregivers as an (unpaid) resource

Although our interviewees started caregiving in different circumstances, many described becoming a caregiver as a gradual process or as a ‘natural’ role, which fits with the view of caregivers as a resource. In fact, few realized from the outset that the tasks they were performing were defined as caregiving in the health system. As Joanne states:

*“I think that a caregiver doesn’t first of all doesn’t see themselves as a caregiver initially. You start off with good intentions of doing something that needs to be done or helping or you know because you love that person, you have a link with that person, a strong one; you’re concerned about them, you want to help”. (Joanne, 46 yrs old, cares for mother)*

Most participants lacked prior caregiving experience, and all of our respondents reported a transition point where they realized that they were no longer simply “lending a helping hand”, especially when the care became more intense, specialized or frequent. This transition appeared to be a key moment in caregivers’ trajectories, leading to considerations for their role within the broader health system, as well as a factor in seeking help and additional resources, such as additional home care resources, physical adaptations to their home, or access to support groups. Yet as one participant stated:

“A *lot of challenges … but it’s not something that you apply for, you sort of fall into it and it’s like on the job training but nobody has the manual”.* David (68 yrs. old, caring for wife)

#### Perceived challenges

Caregivers reported limitations and struggled to continue in their role, to find information, and to be recognized by the healthcare system. Inadequate informational support was most commonly mentioned. Some expressed concern about their limited capacity or interest in caregiving; they feared being unable to respond to future needs or to adjust to care transitions and felt that they lacked adequate training to make decisions about medical care or needs. Caregivers were often not sure where to turn for the right information and answers to their questions; they often felt misled by suggestions that there was more help available than there actually was. Several interviewees knew little about services available to them. For example, Deirdre, though a social worker herself, had not thought of homecare until suggested by her counsellor.

### Caregivers as ‘co-workers’

There was wide variation in the experiences and expectations of caregivers in our sample with regard to feeling part of the healthcare team. Several caregivers described a partnership-like relationship, feeling competent and empowered through sharing their own knowledge and information regarding the care recipient. In some cases, as for Mike, caregivers felt like an integral part of the healthcare team:

*“But when we go to see her specialist together, we work as a team together the three of us, the three specialists together”. (Mike, 63 yrs old, caring for wife)*

Caregivers cherished the value of this therapeutic alliance when it occurred. For instance, Drew was willing to drive 3 h to his mother’s physician because of their unique collaboration:

*“So the practical reality of her condition is such that going to a GP’s office is not an option so we have a brilliant family physician, who has moved three times since I’ve been my mother’s guardian, and we continue to see him even though he’s three hours away by car … it’s such a good experience and support ... (to) go after hours when the clinic is closed I couldn’t do that without him making that available to us, which is brilliant”. (Drew, 38 yrs old, caring for mother)*

#### Perceived challenges

Not all caregivers in this study described being engaged and supported as a co-worker. They were negatively affected when healthcare professionals did not take their concerns seriously, when they were excluded from medical decisions and when they were confronted with unrealistic expectations or negative remarks about their caregiver abilities. One key barrier noted by most caregivers was the lack of training to help manage their loved ones at home. Many were unfamiliar with basic minor medical procedures they were expected to carry out, and they were not always aware of the resources that were at their disposal until after a need had arisen. A number of caregivers spoke about their discomfort in being positioned by the health system in roles that they should not and/or could not fulfil. For example, Deirdre was uncomfortable performing certain procedures herself, such as subcutaneous injections, and found it difficult to manage the daily transportation of her husband, with severe COPD, for blood transfusions and intravenous antibiotics. Caregivers felt challenged by their limited knowledge, but at times, also by that of their healthcare team:

“*So we’re educating the doctors but then it’s, it’s hard to be a pioneer because we’re not doctors and so when we push the edges of our knowledge, we’re also pushing the edges of their knowledge, because they haven’t dealt with this before.” (Claire, 37 yrs old, cares for husband)*

Many caregivers questioned their own competency in being responsible for decisions that they believed should have been made by health professionals, or perhaps made together. For example, both Deirdre and Christiane were uncomfortable about their role in decision-making without the support of the healthcare team. Deirdre wanted healthcare professionals to decide when her husband should be admitted to a facility, but was left to tell him that he could no longer stay at home. Christiane needed more help and was not ready for her husband to be admitted to a long-term care institution, but was left without information or support to make a decision.

Lack of recognition for caregivers’ knowledge and expertise was another important challenge. Drew, for example, knew his mother would only be able to undergo a medical intervention if she could have an unlit cigarette in her hands to calm her down. He describes the struggle with the hospital staff to take him seriously and value his knowledge about his mother and her care.

*“Well lo and behold we finally convinced them that if they wanted to get the scan done we were going to have to work together and I think there’s … the professional and clinical environment that says … “We’re professionals at a hospital we know what to do”. And it’s a little bit of discounting going on there that says well let us handle this. It didn’t work so well and really all it took in the end was for her to hold a cigarette in her hand for her a) to have a catheter without flinching, and b) to get an x-ray done. And so that wasn’t a positive experience.” (Drew, 38 yrs old, cares for mother)*

Finally, tension arose for caregivers in a ‘co-worker’ role because, in contrast to health care workers who had professional responsibility for the care recipient, caregivers juggled roles in multiple domains and this created strain:

“*I may not be here all the time as a caregiver, but I’m here all the time as a son and as a supervisor and as an employer – in some respects I guess a director of care from a clinical standpoint, for no other reason (than) the fact that we’ve done this for 34 years now” (Drew, 38 yrs old, cares for mother).*

This interweaving of personal and professional responsibilities was a major challenge for caregivers:

“*And that’s where respite comes in or help or something. Nurses have time off, caregivers don’t.” (Shayna, 66 yrs old, cares for husband)*

### Caregivers as ‘co-clients’

While most caregivers described how their health was negatively affected by the caregiving situation (such as fatigue, stress, and suicidal thoughts), they rarely expected help through the existing care team of the care recipient. Thus, while caregivers talked about the precarious and challenging balancing act of managing their own health needs, their social lives, and their care responsibilities, few saw themselves as co-clients. Yet, several caregivers described key moments when they received professional acknowledgement and/or support for their own needs as caregivers, which were highlighted as key to their wellbeing. For example, Marlyn was finally persuaded to arrange respite care every 3 months once her doctor convinced her to actually do it, which helped alleviate some of the guilt she felt. Fernanda felt great support when she was given ‘permission’ to hand over the care for her mother:

*“I’ve had again some wonderful experiences … when I was at the end of my rope not knowing how I was going to help my mother and (this doctor) said ‘it’s time, you’ve done your part now it’s time for us to do our part.’ I mean how do I ever, ever, ever thank that doctor for … the kindness he showed me?” (Fernanda, 49 yrs old, caring for mother)*

#### Perceived challenges

Many caregivers described substantial health impacts or health risks caused by their situation but had failed to seek support for themselves, for reasons such as lack of time or because they were unsure where to find support or did not feel entitled to such support unless it was offered by a healthcare professional. In some cases, the engulfing nature of the recipients’ needs precluded any care seeking for themselves. For Shayna, having the same health provider as her husband was problematic as she felt that his needs were prioritized, due to his disability, over her own.

### Caregivers as ‘care coordinators’

Caregivers also spoke about dimensions of their work that could not be neatly subsumed within any of the three roles above, and which we have characterized as a ‘care coordinator’ role. Several caregivers described activities related to information gathering, functioning as the primary organizer, researcher or problem solver in determining best courses of action. Linda herself solved the mystery of her husband’s behavioural issues which were related to medication side effects, and she then led the effort to manage them. Others also assumed attitudes and responsibilities akin to that of a care coordinator.

“*… the person needing the care is constantly running into problems trying to get, trying to do something and so you’re always sort of racking your brain trying to figure out well how else can we make this work? What can we do, how can we adapt this, what could I buy?” (Marlyn, 67 yrs old, caring for husband)*

*“You have to organize care. You have to help with some and you have to deal with a lot of processes and procedures, which don’t fit, they don’t connect.” (Mr. Smith, 62 yrs old, caring for mother-in-law)*

#### Perceived challenges

Participants described challenges in having responsibility for navigating what they perceived to be a fragmented care system, and find solutions to manage the changing needs of their care recipients. Mr. Smith referred to the lack of accountability for continuity of care; he himself was the person making the connections between institutions and coordinating transitions and changes in care.

David spoke about making sure his wife was signed up for a clinical trial, organizing transport to and from the study site. Other caregivers described sorting out transitions to a care facility. Lillian and Michael described their challenges in coming to agreement with each other and with the healthcare professionals regarding the best treatment for their son’s epilepsy. Linda, described how her husband called her just in time from a respite facility for essential medication. In Barbara’s father’s case, she wanted to take charge of the situation but was powerless to change the course of action.

*“Uh just before my dad died um he was in the hospital and he was in very bad shape and on oxygen and really not breathing well and, and the hospital I guess was very short of beds and really needed to empty out as many beds as they could; and so wanted to send him home. Well we lived an hour away from the hospital out on a country road. I’m like ‘are you mad?’ ‘My mum’s blind, I’ve got MS’ and I didn’t know who to talk to. … the nurses would agree with me he should not be sent anywhere. And so but ultimately they sent him home and he lasted maybe 4 hours before an ambulance was called to take him back to the hospital and when he got back within days slipped into unconsciousness and you know died sort of a week after that. I didn’t know who to go to, to stop this from happening; I knew it was going to be a disaster. He had huge bed sores on him that needed to be treated and I mean the whole situation was ludicrous...” (Barbara, 68 yrs old, cared for several people)*

Mike describes how he brought his wife straight back to emergency after she was discharged. It was difficult for him and his wife but it needed to be done to make sure she received the right care. Barbara, Mike, Anne and others described unexpected situations where their expertise was not considered, they were unable to influence decision-making, and where they had to find solutions, even if disruptive to the patient and caregiver and costly to the system. Caregivers appeared to bear the costs related to decisions about optimal care arrangements. Being in the role of a specialized caregiver who is responsible for medical tasks at home made caregivers an important resource and partner in the provision of care, but also often elevated them to a role in care management or coordination.

Caregivers described the difficulties in fulfilling this care coordinator role when they had limited authority to make decisions, required additional resources, a deeper knowledge of the system or the healthcare problem, or help with communicating their needs.

## Discussion

In this article, we sought to examine the extent to which caregivers, in a wide range of circumstances, described their experiences in terms that were congruent with Twigg’s typology of caregivers’ roles [12]. We demonstrated that the typology continues to be relevant for caregivers in current contexts; accounts of being situated as a *resource* were more prevalent, in addition to accounts of roles consistent with that of *co-clients* and *co-workers*. There were relatively few participants who described their experiences as a co-client, which is indicative of the challenge for caregivers in receiving attention for their own needs in spite of abundant evidence related to the negative consequences of caregiving. From our analysis, we also identified a fourth role reflecting participants’ descriptions as *care coordinators*.

The range of routine activities assumed by caregivers in this study typically encompassed engagement with multiple roles in fulfilling day to day responsibilities, and challenges were encountered particular to each type of role. In the absence of changes in policy and practice that respond to caregivers’ needs, the sustainability of their (voluntary) contributions to care, and consequences related to their own health, will ultimately affect the quality, outcomes and overall costs of care at individual and systems levels.

Twigg’s framework [12] highlights the variable character of the relationship of agencies or healthcare organizations towards caregivers; agencies typically relate first to caregivers as a resource to help care for the recipient for which they (the agencies or healthcare professionals) have no formal obligations in terms of care. A recent Canadian study [30], demonstrated the potential value of using a formal assessment tool for caregivers – the Caregivers’ Aspirations, Realities and Expectations – C.A.R.E. Tool, a questionnaire type instrument intended for completion by a healthcare professional with a caregiver. The results indicate that the process of completing the assessment together could encourage professionals to view caregivers as persons in their own right, who have independent but related needs for health and social care, and who are equipped with knowledge to contribute to patient care. Caregivers valued the assessment, which encouraged them to ‘take stock’ of their situations and to build positive relationships with their care professionals.

As co-workers, caregivers move into the intersection between the formal and informal sectors, as per Twigg’s framework. Guberman and Maheu [25] describe how the co-worker perspective requires a move away from the traditional hierarchical relationship to one of co-operation, complementarity and reciprocity that enhances the integration of professional and caregiver expertise. Most caregivers in our study described experiences of feeling like or acting as a co-worker, but this was often met with limited levels of support or recognition. And yet all caregivers in this study talked about being expected to make decisions and perform tasks of a medical nature for which they often felt ill-equipped and poorly supported. The situation of co-operation often failed to materialise for the caregivers in our study.

We argue that the ‘care coordinator’ role warrants separate consideration in future typologies given the descriptions shared by caregivers regarding actions to arrange and coordinate care, as well as navigate complex, poorly integrated care systems, often without support from a broader team. Although it could be argued that this overlaps somewhat with the ‘resource’ and ‘co-worker’ roles, this distinction came out strongly in our data, and may be a reflection of the structural silos and challenges inherent in navigating health systems. Unlike patients, caregivers typically do not become part of a structured care plan where they receive advice and guidance on how to best care for the patients. There are uncertainties about whose responsibility it is to ensure that caregivers are well informed and supported, not only as support for themselves but also to ensure the quality of care provided to the care recipient. Caregivers in our study described care responsibilities that fall in ‘grey’ areas, where it is unclear where the responsibility of the caregiver stops and that of the healthcare professional starts. Caregiver organizations have largely tried to fill the gap for support and advice but it could be argued that certain caregiver guidance and support should be formalized within the health care system.

With caregivers as co-clients, the caregivers’ concerns, as per the framework, become integrated into those of the agencies’ concerns and are treated as care recipients. Very few caregivers in this study expected to be treated as co-clients; in fact, it appeared to come as a surprise when their needs (over and above that of the client) were acknowledged and suggestions were offered for seeking support. This was often described as an important turning point in their caregiving trajectory, as it typically coincided with periods where the situation was rather dire. It seemed that when healthcare teams ‘planted the seed’ – opening a conversation about available resources and ways to find support – caregivers felt they had ‘permission’ to seek help. This lack of attention to caregivers’ needs warrants further attention in practice and policy; a reluctance to seek care until one’s own needs become urgent, as described by participants in this study within the co-client role, puts both caregivers and care recipients at risk.

In this way, it seems that supporting caregivers can be a way to empower them but this may not align with providers’ assumptions about empowerment. A qualitative study, also conducted in Canada, unpacked descriptions of family caregiver empowerment, from the perspectives of homecare managers and leaders [31]. While many shared that educating caregivers, involving them in decision making/ care planning and articulating the caregiver role as worthwhile was empowering for caregivers (particularly during times of crisis) others felt that reliance on health system resources was *disempowering* and were more in favor of supporting self-care. This finding speaks to the importance of unpacking the provider/manager and leader perspectives and assumptions about their roles and goals in supporting caregivers.

Adopting a relationship-centered approach (emphasizing the importance of partnerships between patients, caregivers, and healthcare providers as a central focus for health care delivery), as described by Beach and Inui [32] and developed as the ‘Senses Framework’ by Nolan et al. [33], could help to shape health system responses to the challenges shared by caregivers in our study. Relationship-centered care rests on four foundational principles: of forming and sustaining genuine relationships; being aware of personal biases and honoring the uniqueness of the person; reciprocating; being emotionally present and showing empathy. Such an approach would recognize caregivers as core, valued, members of the team, and create the space for them to express and learn about the care and support that they are entitled to receive.

### Limitations and strengths

Even though the definitions of the three roles presented by Twigg were relatively clear, the delineation between these categories became less clear in the analysis of the caregivers’ narratives. From the relatively small number of narratives collected in our study, we found that caregivers’ roles and responsibilities as described by them were intertwined in a web of different resources, interactions, expectations and needs. Most of our respondents reported enacting at least one, and often all four, roles throughout their caregiving experience. The particular strengths of this study are in the approach for our analysis and contributions to the existing literature on this topic to help advance the conceptual and practical notions of caregiving and its consequences from a broader health and social perspective.

### Research & Practice Improvement Implications

The findings in this study point to the need for further investigation and evidence-informed practice change in at least two areas of enquiry – first, that of the caregiver role as *care coordinator*, to help reduce the substantive burdens associated with navigating complex health and social care systems [34]. Future work should complement the emerging research in areas such as empowering caregivers in this role through educational and practical support services [35], technological advances, such as the use of electronic health tools for caregivers [36] or virtual hospital-to-home communication solutions [37]. More evidence-informed, practical solutions are needed at the levels of system, service and care that offer tangible support across a range of caregiver abilities, roles and contexts. Second, more evidence-based solutions are needed to support caregivers as *co-workers*, as systems transition to more patient-centered approaches, such as how to engage caregivers effectively as members of the care team, or engage with families to co-design services and quality improvements, or to promote shared decision-making [38].

Friedman & Tong developed a framework for Integrating Family Caregivers into the Health Care Team [39]. In this US-based study they conducted a literature review on the role of family caregivers in the coordination of care and key informant interviews with payers, caregivers and care providers to better understand barriers and facilitators to care coordination. They identified 6 areas of focus for policy and practice improvement: identify and record information on family caregivers (as this information is often missing from patient records and caregivers may not self-identify); incentivize providers to engage with caregivers; invest in supports for caregivers; expand access for care coordinators to support caregivers; implement caregiver training programs and develop and improve access to technologies that improve caregiver-provider information exchange.

### Policy implications

Several barriers identified in this study are related to policy or systemic constraints, particularly in the context of co-client and co-worker roles, given limited resources for caregivers and fragmented care contributing to what has been termed the structural burden of caregiving [40]. In Germany [41], where there is a deep-seated culture of familial care ([42], p.123), government policies and programs recognize caregiving as a form of service substitution and provide dedicated resources for caregivers. This includes a cash benefit which, though minimal, offers a strong financial incentive for informal care ([43], p.39) so that even after two decades, the majority of the elderly continue to choose cash payments over in-kind services [44]. As such, caregivers are treated in many respects as co-workers, earning income and receiving social benefits in recognition of their contribution. However, from the perspective of caregivers in this study, similar benefits would go a long way in alleviating some of the most common challenges of being a co-worker, and, in some ways, a co-client.

Similar policy changes in Canada are emerging. In 2014, the Compassionate Care Benefit was introduced for caregivers of people with a serious medical condition who are in their last 6 months of life [45]. Several federal and provincial tax credits also currently exist for caregivers of children and adults with medical conditions and disabilities [46]. However, these financial aids provide only short-term relief and many caregivers are unaware of their eligibility and do not know how to access these supports [4]. Further, in 2011, the Manitoba government instituted the Caregiver Recognition Act, becoming the first Canadian province to formally recognize and commit to increased support for caregivers [47]. Such policy change is necessary, but without changes in how healthcare professionals and organizations value and integrate caregivers as members of the care team, it is insufficient in addressing the barriers experienced by caregivers across their various roles. In the more recent context of the COVID-19 pandemic, the role of caregivers as co-workers and as a co-resource in care has been made abundantly clear, stimulating advances in policy and practice that acknowledge them as ‘essential partners in care’ given evidence of the consequences of interference in their contributions at home and within institutions [48].

## Conclusions

In conclusion, our analysis provides further evidence that caregivers continue to feel invisible and lack appropriate support for their multiple roles [6, 49]. This is despite promising changes in policy and practice to support caregivers in certain jurisdictions [50]. We have suggested an additional role, that of ‘care coordinator’, be formally recognized in designing such supports for those navigating complex and fragmented care. A deeper understanding of the different roles and challenges associated with caregiving can contribute to the design and implementation of policies and services that would support their contributions and choices as integral members of the care team.

Listening to caregivers’ experiences can help us understand and address their needs to optimize experiences for the care recipient as well as for the caregiver. We heard that caregivers are largely unrecognized co-workers and care coordinators (who should also be cared for as co-clients), while the system relies on them as a ‘natural’ resource.

In rethinking our social contract with caregivers, we can draw upon emergent research, progressive policies and innovative practices to recognize their critical roles in human and economic terms within healthcare systems.

## Data Availability

The datasets generated and/or analysed during the current study are not publicly available due to privacy and ethical obligations but may be available from the corresponding author on reasonable request.

## References

[1] Laidsaar-Powell R, Butow P, Bu S, Fisher A, Juraskova I (2016). Attitudes and experiences of family involvement in cancer consultations: a qualitative exploration of patient and family member perspectives. Support Care Cancer.

[2] MacDonald B-J, Wolfson M, Hirdes J. The future cost of long-term care in Canada. Ryerson University: National Institute on Ageing; 2019.

[3] Fast J, Institute for Research on Public Policy. Caregiving for older adults with disabilities: present costs, future challenges. Montreal, Que.: Institute for Research on Public Policy; 2015 Dec [cited 2020 Jun 12]. Report No.: 58. Available from: http://www.deslibris.ca/ID/248569

[4] Kuluski K, Peckham A, Gill A, Arneja J, Morton-Chang F, Parsons J, et al. “You’ve got to look after yourself, to be able to look after them” a qualitative study of the unmet needs of caregivers of community based primary health care patients. BMC Geriatr. 2018;18(1). Available from: https://bmcgeriatr.biomedcentral.com/articles/10.1186/s12877-018-0962-5 10.1186/s12877-018-0962-5 PMC6233534 30419819

[5] Lloyd P (2004). Reconceptualising work with “carers”: new directions for policy and practice. Br J Soc Work.

[6] Ormel I, Law S, Abbott C, Yaffe M, Saint-Cyr M, Kuluski K, Josephson D, Macaulay AC (2017). When one is sick and two need help: caregivers’ perspectives on the negative consequences of caring. Patient Exp J.

[7] Sinha M. Portrait of caregivers. Statistics Canada: Social and Aboriginal Statistics Division; 2013 [cited 2020 Jun 12]. Report No.: 89–652-X–001. Available from: https://www.deslibris.ca/ID/240440

[8] Ansello EF, Rosenthal C (2007). Hidden costs and invisible contributions in family caregiving: an introduction. Can J Aging Rev Can Vieil.

[9] Queluz FNFR, Kervin E, Wozney L, Fancey P, McGrath PJ, Keefe J (2020). Understanding the needs of caregivers of persons with dementia: a scoping review. Int Psychogeriatr.

[10] Keefe J, Institute for Research on Public Policy. Supporting caregivers and caregiving in an aging Canada. Montreal, Que.: Institute for Research on public policy; 2012 [cited 2020 Jun 12]. Available from: https://www.deslibris.ca/ID/230409

[11] Andrew MK, Dupuis-Blanchard S, Maxwell C, Giguere A, Keefe J, Rockwood K (2018). Social and societal implications of frailty, including impact on Canadian healthcare systems. J Frailty Aging.

[12] Twigg J (1989). Models of carers: how do social care agencies conceptualise their relationship with informal carers?. J Soc Policy.

[13] Twigg J, Atkin K (1994). Carers perceived: policy and practice in informal care.

[14] Feely A, Lix LM, Reimer K (2017). Estimating multimorbidity prevalence with the Canadian chronic disease surveillance system. Health Promot Chronic Dis Prev Can.

[15] Barnett K, Mercer SW, Norbury M, Watt G, Wyke S, Guthrie B (2012). Epidemiology of multimorbidity and implications for health care, research, and medical education: a cross-sectional study. Lancet.

[16] Van Lerberghe W (2008). The world health report 2008: primary health care (now more than ever).

[17] Boyd CM, Fortin M (2010). Future of multimorbidity research: how should understanding of multimorbidity inform health system design?. Public Health Rev.

[18] Soklaridis S, Ravitz P, Adler Nevo G, Lieff S (2016). Relationship-centred care in health: a 20-year scoping review. Patient Exp J..

[19] MacCourt P (2013). Family caregivers advisory committee, Mental Health Commission of Canada. National guidelines for a comprehensive service system to support family caregivers of adults with mental health problems and illnesses.

[20] Wilson M, Gauvin F, Ploeg J (2014). Citizen brief: improving care and support for unpaid caregivers in Ontario.

[21] Armstrong P, Kits O, Grant K, Amaratunga C, Armstrong P, Boscoe M, Pederson A, Kay W (2004). One hundred years of caregiving. Caring for/caring about: women, home care, and unpaid caregiving.

[22] Ward-Griffin C, McKeever P (2000). Relationships between nurses and family caregivers: partners in care?. Adv Nurs Sci.

[23] Stajduhar KI, Nickel DD, Martin WL, Funk L (2008). Situated/being situated: client and co-worker roles of family caregivers in hospice palliative care. Soc Sci Med.

[24] Glendinning C, Mitchell W, Brooks J (2015). Ambiguity in practice? Carers’ roles in personalised social care in England. Health Soc Care Community.

[25] Guberman N, Maheu P (2002). Conceptions of family caregivers: implications for professional practice. Can J Aging Rev Can Vieil..

[26] Wiles J (2003). Informal caregivers’ experiences of formal support in a changing context. Health Soc Care Community..

[27] Patton MQ (2002). Qualitative Research & Evaluation Methods.

[28] Hallberg LR-M (2006). The “core category” of grounded theory: making constant comparisons. Int J Qual Stud Health Well-Being.

[29] Walker D, Myrick F (2006). Grounded theory: an exploration of process and procedure. Qual Health Res.

[30] Guberman N, Keefe J, Fancey P. The assessment experience of spousal dementia care-givers: ‘It’s made me realise that I am a person also.’ Ageing Soc 2019;39(11):2443–2464, The assessment experience of spousal dementia care-givers: ‘It's made me realise that I am a person also’, DOI: 10.1017/S0144686X18000557.

[31] Stajduhar KI, Funk L, Wolse F, Crooks V, Roberts D, Williams AM, et al. Principaux aspects de l’autonomie des aidants d’après les chefs de file en matière de soins à domicile : soins palliatifs et maladie chronique 2011;43(3):18.

[32] Beach MC, Inui T (2006). Relationship-centered care research network. Realtionship-centered care: a constructive reframing. J Gen Intern Med.

[33] Nolan M, Brown J, Davies S, Nolan J, Keady J. The Senses Framework: Improving Care for Older People Through a Relationship-Centred Approach. 2006. Available from: http://shura.shu.ac.uk/280/1/pdf_senses_framework_report.pdf

[34] Funk L. Relieving the burden of navigating health and social services for older adults and caregivers. Montreal: Institute for Research on Public Policy; 2019 IRRP Study No 73.

[35] Bruening R, Sperber N, Miller K, Andrews S, Steinhauser K, Wieland GD, Lindquist J, Shepherd-Banigan M, Ramos K, Henius J, Kabat M, van Houtven C (2020). Connecting caregivers to support: lessons learned from the va caregiver support program. J Appl Gerontol.

[36] Giroux D, Tremblay M, Latulippe K, Provencher V, Poulin V, Giguere A, Dubé V, Sévigny A, Guay M, Ethier S, Carignan M (2019). Promoting identification and use of aid resources by caregivers of seniors: co-design of an electronic health tool. JMIR Aging.

[37] Smith PD, Martin B, Chewning B, Hafez S, Leege E, Renken J, Smedley Ramos R (2018). Improving health care communication for caregivers: a pilot study. Gerontol Geriatr Educ.

[38] Hamann J, Heres S (2019). Why and how family caregivers should participate in shared decision making in mental health. Psychiatr Serv.

[39] Friedman E, Tong P. A Framework for integrating family caregivers into the health care team. RAND Corporation; 2020 [cited 2021 Jan 15]. Available from: https://www.rand.org/pubs/research_reports/RRA105-1.html

[40] Taylor MG, Quesnel-Vallée A (2017). The structural burden of caregiving: shared challenges in the United States and Canada. The Gerontologist.

[41] Quesnel-Vallée A, Burau V, Jahagirdar D, Graham E, Reiter-Campeau S (2014). Continuing care coverage in Canada: comparative study of long-term care in Australia and Germany.

[42] Theobald H, Hampel S, Ranci C, Pavolini E (2013). Radical institutional change and incremental transformation: long-term care insurance in Germany. Reforms in long-term care policies in Europe: investigating institutional change and social impacts.

[43] Schunk M, Glendinning C (1998). The social insurance model of care for older people in Germany. Rights and realities: comparing new developments in long-term care for older people.

[44] Eichler M, Pfau-Effinger B (2009). The ‘consumer principle’ in the care of elderly people: free choice and actual choice in the german welfare state. Soc Policy Adm.

[45] Service Canada. Employment insurance: in difficult times: compassionate care benefits. Service Canada. 2014; Available from: https://www.cancer.ca/~/media/cancer.ca/AB/get%20involved/take%20action/FederalCompassinateCareBenefitProgarm-AB.pdf?la=en.

[46] Government of Canada. Tax credits for caregivers. 2018. Available from: https://www.canada.ca/en/financial-consumer-agency/services/caring-someone-ill/tax-credit-caregiver.html

[47] Manitoba Health. Healthy living and seniors. In: Caregiver recognition act: report for the period, 2013–2015. Winnipeg, Manitoba; 2013 p. 11. Available from: https://www.gov.mb.ca/health/documents/caregiver_recognition_act_report.pdf

[48] Munshi L, Evans G, Razak F. The case for relaxing no-visitor policies in hospitals during the ongoing COVID-19 pandemic. Can Med Assoc J. 2021;193(4):E135–E137. 10.1503/cmaj.202636. 10.1503/cmaj.202636 PMC7954559 33355198

[49] Kokorelias KM, Lu FKT, Santos JR, Xu Y, Leung R, Cameron JI (2020). “Caregiving is a full-time job” impacting stroke caregivers’ health and well-being: a qualitative meta-synthesis. Health Soc Care Community..

[50] Scheil-Adlung X. Long term care protection for older persons: a review of coverage deficits in 46 countries. Geneva: UNECE Working Group on Ageing; 2015. Available from: https://www.unece.org/fileadmin/DAM/pau/age/WG8/Presentations/4b_Long_Term_Care_Working_Group_on_Ageing.pdf

